# Fermentation and Metabolic Pathway Optimization to De Novo Synthesize (2*S*)-Naringenin in *Escherichia coli*

**DOI:** 10.4014/jmb.2008.08005

**Published:** 2020-08-21

**Authors:** Shenghu Zhou, Tingting Hao, Jingwen Zhou

**Affiliations:** 1 National Engineering Laboratory for Cereal Fermentation Technology, Jiangnan University, Wuxi, Jiangsu 2422, P.R. China; 2 Jiangsu Provincial Research Center for Bioactive Product Processing Technology, Jiangnan University, Wuxi, Jiangsu 141, P.R. China

**Keywords:** L-tyrosine, *p*-coumaric acid, dynamic regulation, flavonoids, temperature-shift

## Abstract

Flavonoids have diverse biological functions in human health. All flavonoids contain a common 2-phenyl chromone structure (C6-C3-C6) as a scaffold. Hence, in using such a scaffold, plenty of highvalue-added flavonoids can be synthesized by chemical or biological catalyzation approaches. (2*S*)-Naringenin is one of the most commonly used flavonoid scaffolds. However, biosynthesizing (2*S*)-naringenin has been restricted not only by low production but also by the expensive precursors and inducers that are used. Herein, we established an induction-free system to de novo biosynthesize (2*S*)-naringenin in *Escherichia coli*. The tyrosine synthesis pathway was enhanced by overexpressing feedback inhibition-resistant genes (*aroG*^fbr^ and *tyrA*^fbr^) and knocking out a repressor gene (*tyrR*). After optimizing the fermentation medium and conditions, we found that glycerol, glucose, fatty acids, potassium acetate, temperature, and initial pH are important for producing (2*S*)-naringenin. Using the optimum fermentation medium and conditions, our best strain, Nar-17LM1, could produce 588 mg/l (2*S*)-naringenin from glucose in a 5-L bioreactor, the highest titer reported to date in *E. coli*.

## Introduction

Flavonoids are value-added nutritional chemicals that have diverse biological functions; they are antibacterial, anti-atherosclerotic, anti-carcinogenic, and they protect the liver [1, 2]. Under the current industrial production scale, flavonoids are extracted mainly from plant tissues [3, 4]. However, the low content in plant tissues results in a low titer and a high price for flavonoids that cannot satisfy the demands of the market [5]. Furthermore, the harmful solvents and extreme conditions needed in the extraction process also hinder the application of plant extraction [3]. Hence, it is necessary to develop a flavonoid production process that is environmentally friendly and has high efficiency.

Although there are thousands of flavonoids with significantly different structures, all of them contain a common 2-phenyl chromone structure (C6-C3-C6) as a scaffold [6]. Hence, any desired flavonoid could be synthesized from a flavonoid scaffold by either enzymatic or chemical catalyzation methods. In this regard, the achievement of large-scale production by a microbial fermentation approach using flavonoid scaffolds is important for green production. (2*S*)-Naringenin is one of the most common flavonoid scaffolds and has many nutritional and pharmaceutical functions in human health [1, 7, 8]. *p*-Coumaric acid and tyrosine are important precursors of (2*S*)-naringenin. Therefore, some researchers have used them to produce (2*S*)-naringenin. Using tyrosine as a substrate, Wu *et al*. fine-tuned the metabolic flux of the malonyl-CoA synthesis pathway through antisense RNA [9] and CRISPR interference [10] approaches, obtaining 391 mg/l and 421.6 mg/l of (2*S*)-naringenin in *Escherichia coli*, respectively. In *Saccharomyces cerevisiae*, Lyu *et al*. obtained ~90 mg/l of naringenin from tyrosine after optimizing the malonyl-CoA and naringenin synthesis pathway [11]. Compared with tyrosine, *p*-coumaric acid as a substrate could significantly improve the titer of (2*S*)-naringenin. In recent reports, Gao *et al*. systemically optimized the synthesis pathway of (2*S*)-naringenin in *S. cerevisiae*, resulting in titers of 648.63 mg/l and 1.21 g/l by using *p*-coumaric acid as a substrate [12, 13]. These researchers have shown that engineered microbes are capable of producing flavonoids on a large scale.

However, the added tyrosine and *p*-coumaric acid increase the cost of fermentation. Hence, cheaper carbon sources are preferred in the fermentation process. To synthesize (2*S*)-naringenin from glucose, Wu *et al*. overexpressed 3-deoxy-D-arabinoheptulosonate-7-phosphate (DAHP) synthase (*aroG*^fbr^) and chorismate mutase/prephenate dehydrogenase (*tyrA*^fbr^) in *E. coli*. This released the feedback inhibition by tyrosine to finally yield 100.64 mg/l (2*S*)-naringenin [14]. Furthermore, Raman *et al*. obtained 61 mg/l of (2*S*)-naringenin from glucose in *E. coli* by a biosensor-based directed evolution strategy [15]. According to recent reports, *Yarrowia lipolytica* generated a higher (2*S*)-naringenin titer than *E. coli*. Lv *et al*. optimized the biosynthetic pathway of (2*S*)-naringenin by multi-module strategy and obtained a 252.4 mg/l titer from glucose [16]. Wei *et al*. obtained a 715.3 mg/l titer in *Y. lipolytica* by using a xylose-responsive biosensor to control expression of the (2*S*)-naringenin synthesis pathway [17]. In this process, xylose simultaneously acted as a substrate and inducer. Recently, Palmer *et al*. broke this record by applying a β-oxidation-related strategy in *Y. lipolytica*. They generated 898 mg/l of (2*S*)-naringenin from glucose, the highest titer reported to date in any host [18].

**Fig. 1:**
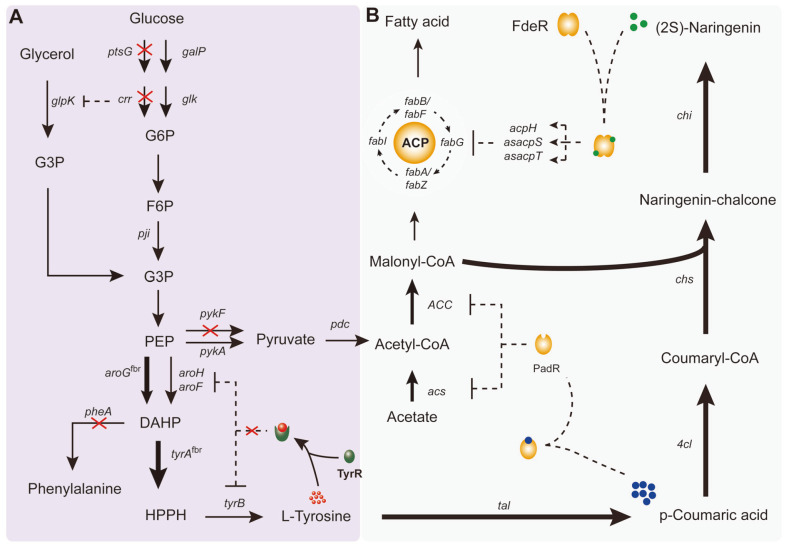
The de novo biosynthesis pathway for (2*S*)-naringenin. (**A**) Metabolic engineering to enhance the metabolic flux from glucose to L-tyrosine. The red crosses represent genes that were deleted. The bold arrows represent genes that were overexpressed. The thin arrows represent genes that were natively expressed. (**B**) The dynamic regulation network of Mut-17, which was constructed in our previous study [19]. (2*S*)-Naringenin synthesis pathway was constitutively overexpressed [7]. Hence, (2*S*)-naringenin could accumulate with cell growth. The accumulated (2*S*)-naringenin activates activator FdeR [37] to repress the fatty acid synthesis pathway to reduce the consumption of malonyl-CoA. Meanwhile, the repressor PadR [38] can be inactivated when *p*-coumaric acid accumulates, resulting in the expression of acetyl-CoA synthase (*acs*) and acetyl-CoA carboxylase (*ACC*) to enhance the biosynthesis of malonyl-CoA. Compound annotation: glyceraldehyde 3-phosphate (G3P); phosphoenolpyruvate (PEP); glucose-6-phosphate (G6P); fructose-6-phosphate (F6P); 4 hydroxyphenylpyruvate (HPPH); Gene or enzyme annotation: glycerol kinase (*glpK*); pyruvate kinase I/II (*pykF*/*pykA*); tyrosine repressor (TyrR); acyl carrier protein (ACP); tyrosine aminotransferase (*tyrB*); DAHP synthase (*aroG*/*aroH*/*aroF*); chorismate mutase/prephenate dehydratase (*pheA*); glucose-specific IIACB component (*ptsG*/*crr*); galactose permease (*galP*); glucokinase (*glk*); phosphodiesterase (*acpH*); anti-sense RNA of holo-ACP synthase (*asacpS*) and holo-ACP synthase 2 (*asacpT*); tyrosine ammonia-lyase (*tal*), 4-coumarate: CoA ligase (*4cl*), chalcone synthase (*chs*), chalcone isomerase (*chi*).

According to current reports, the titer of (2*S*)-naringenin from *E. coli* as host has usually been far lower than from either *S. cerevisiae* or *Y. lipolytica*. However, the various superior properties of *E. coli*, such as its easy engineering and fast growth, make it one of the best hosts for (2*S*)-naringenin production. Hence, it is important to further improve the titer of (2*S*)-naringenin through de novo biosynthesis. In this study, we established an induction-free system in our previously constructed dynamic regulated *E. coli* strain, which can achieve automatic balancing of (2*S*)-naringenin production, *p*-coumaric acid accumulation, and cell growth (Figs. 1A and 1B) [19]. To do this, we first optimized the fermentation conditions (pH and temperature) and medium contents (carbon sources, nitrogen source, and MnCl_2_). Then, we optimized the tyrosine synthesis pathway for de novo biosynthesis of (2*S*)-naringenin. The optimum strain produced 588 mg/l (2*S*)-naringenin from glucose in a 5-L bioreactor. This is the highest titer reported to date in *E. coli*.

## Materials and Methods

### Strains, Medium, and Culture Conditions

*E. coli* JM109 and *E. coli* BL21 (DE3) were used as hosts for plasmid construction and pathway expression, respectively. Luria-Bertani broth (LB) medium was used to enrich cells for plasmid construction or fermentation seed culture. MOPS minimal medium [20] (supplemented with 5 g/l D-glucose and 4 g/l NH_4_Cl) was used as the initial medium for fermentation optimization. The L-tyrosine was dissolved in sodium hydroxide solution to a final concentration of 300 mM. Then, 1% of L-tyrosine solution was added in the medium to obtain the 3 mM final concentration. In the fermentation optimization process, strains were cultured at 30°C and 220 rpm. Shake flask fermentation was performed at 220 rpm with 3% of seed cultures under optimum fermentation conditions and medium. Ampicillin (100 μg/ml), streptomycin (50 μg/ml), chloramphenicol (34 μg/ml), and kanamycin (50 μg/ml) were added to the media as required. The details of the strains and plasmids used in this study are listed in Table 1.

### Fermentation Optimization

Fermentation seeds were cultured for 12 h in LB medium and used to optimize the fermentation conditions and medium components. In order to optimize the fermentation temperature, after incubation, the strains were cultured at 37°C for different times (2, 4, 7, and 10 h) for cell growth, and then transferred to 30°C, 25°C, and 20°C for (2*S*)-naringenin production. The titers of (2*S*)-naringenin and *p*-coumaric acid were measured after 48 h of fermentation. Furthermore, the initial pH value (5.0, 6.0, 7.0, and 8.0) was also optimized. All these optimizations were performed in deep 24-well plates. Triplicate biological replicates were performed in every fermentation experiment.

Different concentrations of carbon and nitrogen sources were added in the original MOPS minimal medium in deep 24-well plates to optimize the medium components. Specifically, the optimum concentrations of KAc (1.2, 2.5, 5, and 7.5 g/l), peptone (2.5, 5, 10, and 15 g/l), glucose (5.5, 10, 15, and 20 g/l), ammonia chloride (6.5, 9, 14, and 19 g/l), yeast extract (2.5, 5, 10, and 15 g/l), glycerol (2.5, 5, 10, and 15 g/l), myristic acid (2.5, 5, 10, and 15 g/l), palmitic acid (2.5, 5, 10, and 15 g/l), stearic acid (2.5, 5, 10, and 15 g/l), and cis-11-octadecenoic acid (2.5, 5, 10, and 15 g/l) in the MOPS minimal medium were investigated. The titers of (2*S*)-naringenin and *p*-coumaric acid were measured after 48 h of fermentation in the various modified MOPS media. Then, the components of the final fermentation medium were obtained by a combination of the optimum carbon and nitrogen sources. The fed-batch fermentation was performed in a 5-L bioreactor. Nar-17LM1 strain was used for fed-batch fermentation in the optimum medium at pH 7.0, 1 vvm aeration, and 400 rpm stirring. Then, 100 ml of overnight cultured Nar-17LM1 was added in 3 L of fermentation medium. When the OD_600_ of the cultures reached 3.0 with culture at 37°C, the temperature was shifted to 25°C for (2*S*)-naringenin production. In this process, 30 ml of 500 g/l glucose was added to the bioreactor for 60 h, 72 h, 90 h, and 96 h for fermentation.

### Plasmid Construction

The promoters P_*talB*_ and P_*glpD*_ were amplified from the genome of *E. coli* K12 MG1655 by using the primer pairs of P_*talB*_-*aroG*-F/P_*talB*_-*aroG*-R and P_*glpD*_-*tyrA*-F/P_*glpD*_-*tyrA*-R, respectively. Promoter P_*yjiY*_ was also amplified from the genome of *E. coli* K12 MG1655 using the primer pairs of P_*yjiY*_-*aroG*-F/P_*yjiY*_-*aroG*-R and P_*yjiY*_-*tyrA*-F/P_*yjiY*_-*tyrA*-R. pMD-*tyrA*^fbr^-*aroG*^fbr^ was linearized by whole plasmid PCR using the primer pairs of *tyrA*-F/*tyrA*-R, and then ligated with promoters P_*yjiY*_ and P_*glpD*_ by the Gibson assembly approach to obtain the plasmids pMD-P_*yjiY*_-*tyrA*^fbr^-*aroG*^fbr^ and pMD-P_*glpD*_-*tyrA*^fbr^-*aroG*^fbr^, respectively. Furthermore, pMD-P_*yjiY*_-*tyrA*^fbr^-*aroG*^fbr^ and pMD-P_*glpD*_-*tyrA*^fbr^-*aroG*^fbr^ were linearized by whole plasmid PCR using the primer pairs of *aroG*-F/*aroG*-R and then ligated with promoters P_*yjiY*_ and P_*talB*_ by the Gibson assembly approach to obtain the plasmids pMD-P_*yjiY*_-*tyrA*^fbr^-P_*yjiY*_-*aroG*^fbr^, pMD-P_*yjiY*_-*tyrA*^fbr^-P_*talB*_-*aroG*^fbr^, pMD-P_*glpD*_-*tyrA*^fbr^-P_*yjiY*_-*aroG*^fbr^, and pMD-P_*glpD*_-*tyrA*^fbr^-P_*talB*_-*aroG*^fbr^. Then, the promoter-*tyrA*^fbr^-promoter-*aroG*^fbr^ DNA fragments were amplified from these four plasmids, and ligated with the XbaI/BamHI digested plasmid pCDM-P_ssrA_-UTR_rpsT_-CHS-PUTR_glpD_-CHI by the Gibson assembly approach to obtain the plasmids pCDM-P_ssrA_-UTR_rpsT_-*chs*-PUTR_glpD_-*chi*-P_*yjiY*_-*tyrA*^fbr^-P_*talB*_-*aroG*^fbr^, pCDM-P_ssrA_-UTR_rpsT_-*chs*-PUTR_glpD_-*chi*-P_*yjiY*_-*tyrA*^fbr^-P_*yjiY*_-*aroG*^fbr^, pCDM-P_ssrA_-UTR_rpsT_-*chs*-PUTR_glpD_-*chi*-P_*glpD*_-*tyrA*^fbr^-P_*yjiY*_-*aroG*^fbr^, and pCDM-P_ssrA_-UTR_rpsT_-*chs*-PUTR_glpD_-*chi*-P_*glpD*_-*tyrA*^fbr^-P_*talB*_-*aroG*^fbr^. High-fidelity DNA polymerase and DNA restriction enzymes were purchased from Takara (Dalian, China). The details of the primers used in this study are listed in Table 2.

### Analysis Methods

To quantitatively analyze the titers of (2*S*)-naringenin and *p*-coumaric acid, we mixed 1 mL of the fermentation samples with an equivalent volume of 100% ethyl alcohol and set it at room temperature for 30 min to lyse the cell wall and dissolve the products. Then, the supernatants of these samples were filtered through 0.22 µm nylon 6 filters after 12,000 ×*g* centrifugation for 5 min. Finally, the filtered supernatants were analyzed by using an Agilent 1100 HPLC system at 290 nm. The degassed mixture of methyl alcohol (41%), phosphoric acid (0.3%), and water (58.7%) was used as the mobile phase after being filtered through 0.22 µm nylon 6 filters. A reverse-phase C18 column (4.6 × 150 mm, Thermo, USA) was used to separate (2*S*)-naringenin and *p*-coumaric acid at 35°C with a constant flow rate of 1 ml/min.

## Results

### Optimization of Medium Components

**Fig. 2:**
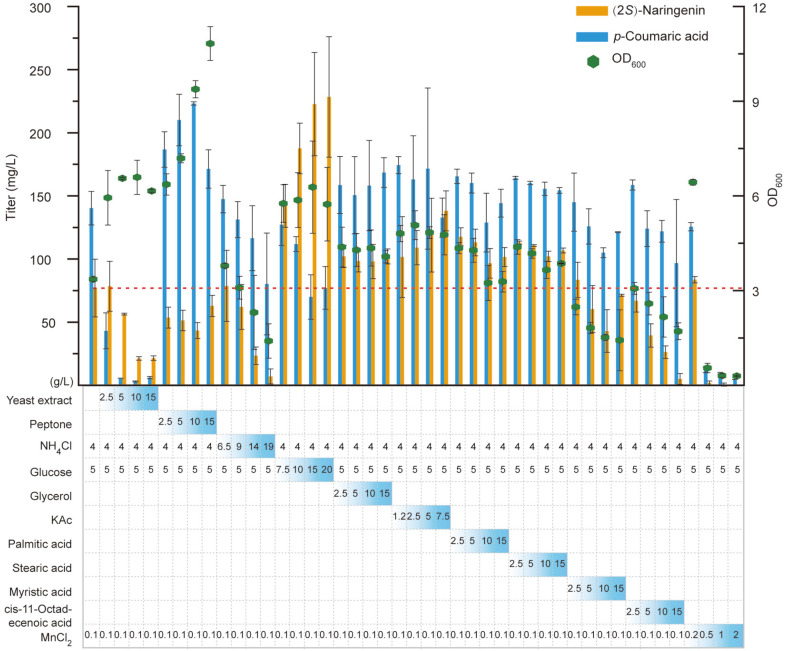
The influence of medium components on growth state, as well as (2*S*)-naringenin and *p*-coumaric acid titer, of Mut-17. Different concentrations of extra nitrogen sources, carbon sources, fatty acids, and MnCl_2_ were added to the MOPS minimal medium. MOPS minimal medium already contains 4 g/l NH_4_Cl, 5 g/l glucose, and 0.1 g/l MnCl_2_. Fermentation in MOPS minimal medium was used as the control. The red dashed line represents the (2*S*)-naringenin titer of the control.

Medium components not only influence cell growth, they are also closely related to the production of (2*S*)-naringenin. To supply optimal growth and production conditions, we first optimized the concentration of different carbon sources, nitrogen sources, fatty acids, and Mn^2+^. In doing so, Mut-17 was used as the producer, and MOPS minimal medium was used as the initial fermentation medium. Because barren MOPS medium is not suited to cell growth, we optimized the carbon sources and nitrogen sources. The results show that yeast extract and peptone significantly improved the growth state of Mut-17, but negatively impacted (2*S*)-naringenin production (Fig. 2). With increasing concentrations of yeast extract and peptone, the titer of (2*S*)-naringenin gradually decreased. However, the titer of *p*-coumaric acid gradually decreased and increased with yeast extract and peptone, respectively. Furthermore, high concentrations of the inorganic nitrogen source NH_4_Cl significantly repressed cell growth and (2*S*)-naringenin production. Hence, 4 g/l NH_4_Cl was the optimum nitrogen source.

Malonyl-CoA is the main limiting factor for (2*S*)-naringenin biosynthesis [21, 22]. Hence, it is necessary to enhance the production of malonyl-CoA at the fermentation level. The direct precursor of malonyl-CoA is acetyl-CoA, whose biosynthesis is closely related to carbon sources and acetate (Fig. 1). With this background, we optimized the concentrations of glucose, glycerol, and potassium acetate (KAc) in the MOPS medium. We found that a high glucose concentration significantly improved the titer of (2*S*)-naringenin, and the highest titer reached 2.96-fold that of the control when 20 g/l glucose was added (Fig. 2). However, 15 g/l glucose almost achieved the highest titer and had the highest final OD_600_. Hence, we used 15 g/l glucose as the optimum concentration. On the other hand, both glycerol and KAc were beneficial for (2*S*)-naringenin production and cell growth. Different glycerol concentrations had no significant influence on (2*S*)-naringenin biosynthesis and cell growth, but higher concentrations of KAc improved them. Overall, the optimum carbon sources were glucose at 15 g/l, glycerol at 2.5 g/l, and KAc at 7.5 g/l.

Malonyl-CoA mainly flows into the native fatty acid pathway. Thus, repressing the metabolic flux of the fatty acid pathway could efficiently enhance the accumulation of malonyl-CoA [23]. Here, the dynamic regulation system of Mut-17 could repress the fatty acid synthesis pathway according to the concentration of (2*S*)-naringenin to enhance the accumulation of malonyl-CoA. Hence, we speculated that adding extra fatty acid would improve cell growth and (2*S*)-naringenin production. To prove this, we added different concentrations of palmitic acid, stearic acid, myristic acid, and cis-11-octadecenoic acid into the MOPS medium for fermentation. The results show that 2.5 g/l each of palmitic acid and stearic acid was the optimum concentration for both cell growth and (2*S*)-naringenin production (Fig. 2). Adding myristic acid and cis-11-octadecenoic acid repressed both cell growth and (2*S*)-naringenin production. On the other hand, acyl carrier protein (ACP) is an important protein in the fatty acid biosynthesis process. Repressing the activity of ACP improved the accumulation of malonyl-CoA. Given that AcpH is an Mn^2+^-dependent phosphodiesterase [24], optimizing the concentration of Mn^2+^ would probably be helpful for fermentation. For this reason, we added different concentrations of MnCl_2_ into the MOPS medium and found that high concentrations significantly repressed cell growth (Fig. 2). Finally, the optimum concentration of MnCl_2_ was identified as 0.1 g/l.

### Optimization of the Fermentation Conditions

**Fig. 3:**
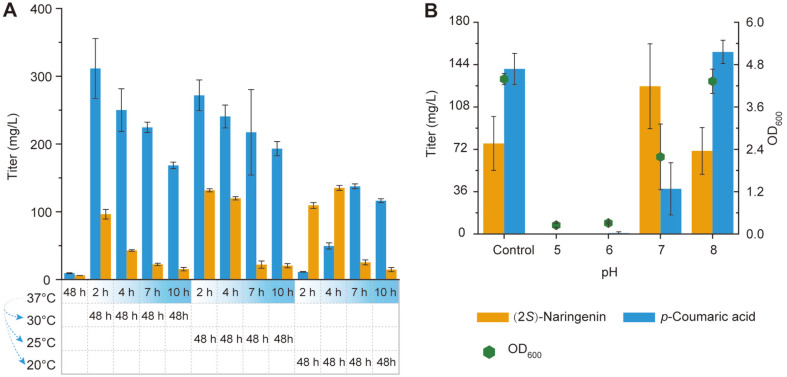
Temperature and pH optimization. (**A**) Temperature-shift strategy to optimize the time and temperature of the growth stage and production stage. (**B**) Initial pH optimization. The control had a natural pH (7.4) of MOPS.

Temperature and pH are important factors that can significantly influence cell growth and pathway enzyme activity. Generally, fermentation requires a set of conditions for cell growth in the early growth stage and then a set of conditions for the production stage. Wherein, temperature not only influenced cell growth but also significantly influenced the activity of enzymes. Furthermore, the enzymes from different origins usually have different optimum catalyzation temperature [25]. With this background, we investigated a temperature-shift fermentation strategy. Mut-17 was cultured at 37°C for different times for cell growth, and then transferred to a lower temperature for (2*S*)-naringenin biosynthesis (Fig. 3A). With increasing culture times at 37°C, the titers of (2*S*)-naringenin and *p*-coumaric acid were significantly decreased. A low temperature in the production stage promoted (2*S*)-naringenin biosynthesis, but reduced the biosynthesis of *p*-coumaric acid (Fig. 3A). This indicates that the optimum catalyzation temperature of TAL was probably different with the downstream enzymes (4CL, CHS, and CHI). Wherein, the activity of downstream enzymes would increase under lower temperature and thus enhanced the conversion from *p*-coumaric acid to (2*S*)-naringenin. Finally, we identified that culturing at 37°C for 2 h and then transferring to 25°C was the optimum temperature-shift fermentation condition. In addition, pH can also influence cell growth and production. Hence, we optimized the initial pH. The results show that a neutral or alkaline pH condition was better than a faintly acid condition (Fig. 3B). The optimum initial pH was identified as 7.0.

**Fig. 4:**
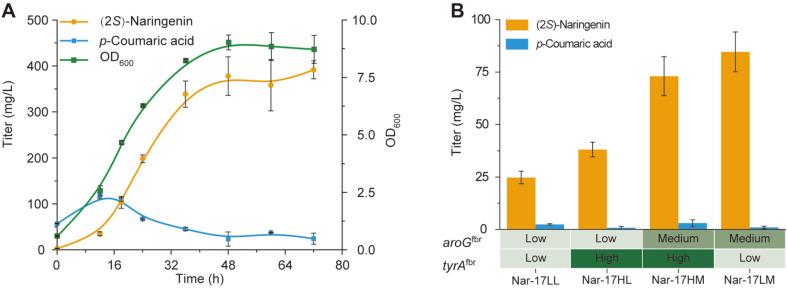
Shake flask fermentation in the optimum medium for (2*S*)-naringenin production from tyrosine (**A**) and glucose (**B**). (**A**) Mut-17 was used for fermentation; (**B**) The expression levels of the anti-feedback inhibition genes (*aroG*^fbr^ and *tyrA*^fbr^) were optimized by promoters with different strengths. 0 h represents the starting time of temperature shifting.

Fermentation was subsequently performed in a shake flask under the above optimum medium and conditions (that is, adding 4 g/l NH_4_Cl, 15 g/l glucose, 2.5 g/l glycerol, 7.5 g/l KAc, 2.5 g/l palmitic acid, and 2.5 g/l stearic acid in MOPS minimal medium, initial pH 7.0, 37°C culture for 2 h and then transfer to 25°C). To maintain a pH higher than 5.6 in the fermentation process, we added 5 g/l CaCO_3_ to the shake flasks. Finally, Mut-17 produced 391 mg/l (2*S*)-naringenin from 3 mM L-tyrosine with 24 mg/l *p*-coumaric acid accumulation (Fig. 4A). This (2*S*)-naringenin titer was 1.6-fold (391 mg/l vs. 251 mg/l) higher than the un-optimized titer in our previous report [19].

### Releasing the Feedback Inhibition for De Novo Synthesis of (2*S*)-Naringenin

In the tyrosine synthesis pathway, *aroG* and *tyrA* could be feedback-repressed by tyrosine. Hence, we overexpressed their anti-feedback inhibition mutants (*aroG*^fbr^ and *tyrA*^fbr^) [14] in Mut-17. Three different strength promoters—P_*yjiY*_ (low strength), P_*talB*_ (medium strength), and P_*glpD*_ (high strength) [26]–were used to control the expression levels of *aroG*^fbr^ and *tyrA*^fbr^. Comparing Nar-17LL with Nar-17-LM and Nar-17HL with Nar-17-HM, we found that the higher expression level of *aroG*^fbr^ yielded a higher (2*S*)-naringenin titer (Fig. 4B). Hence, *aroG*^fbr^ was probably the rate-limiting step for the biosynthesis of tyrosine. The highest titer of (2*S*)-naringenin was 85 mg/l, which was far lower than the titer in Mut-17. We also analyzed the accumulation level of *p*-coumaric acid and tyrosine and found that the accumulation of *p*-coumaric acid was lower than 3 mg/l, and no tyrosine was detected. Such results indicate that the current metabolic flux of the tyrosine biosynthesis pathway could not satisfy the demands of downstream (2*S*)-naringenin biosynthesis.

### Enhancing the Metabolic Flux from Glucose to L-Tyrosine

**Fig. 5:**
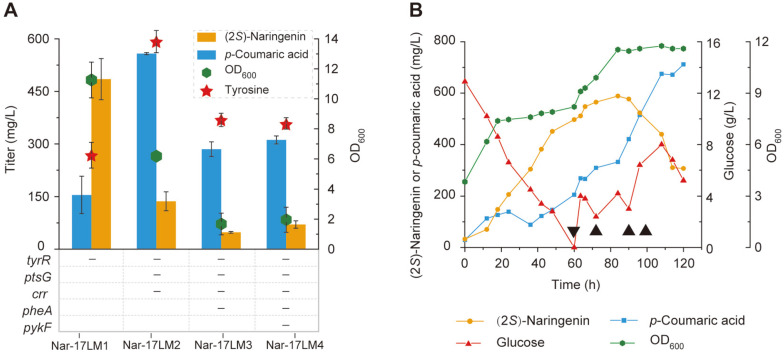
De novo biosynthesis of (2*S*)-naringenin by knockout strains in a shake flask (**A**) and 5-L bioreactor (**B**). (**A**) A minus represents the relative genes that were knocked out; (**B**) Nar-17LM1 was the producer for fed-batch fermentation. Black triangles represent the time points at which 30 mL of 500 g/l glucose was fed. 0 h represents the starting time of temperature shifting.

In order to further improve the metabolic flux of the tyrosine biosynthesis pathway, we introduced the (2*S*)-naringenin synthesis pathway (including pETM-PUTR_trxA_-TAL-PUTR_talB_-4CL, pCDM-P_ssrA_-UTR_rpsT_-*chs*-PUTR_glpD_-*chi*-P_*yjiY*_-*tyrA*^fbr^-P_*talB*_-*aroG*^fbr^, pACM-P_fdeR_-FdeR-P_fdeA-17_-*acpH*-*asacpT*-*asacpS*, and pRSM-PadR-*acs*-*ACC*, see Table 1) into four knockout strains [27] and generated strains of Nar-17LM1, Nar-17LM2, Nar-17LM3, and Nar-17LM4 (Fig. 5A). TyrR is a tyrosine-activated repressor that can repress the expression of *aroH*, *aroF*, and *tyrB* (Fig. 1). After knocking out *tyrR*, the titer of (2*S*)-naringenin improved to 485 mg/l, which was 5.7-fold higher than that of Nar-17-LM. The second knockout target was the phosphotransferase system (PTS) since the function of PTS needs to sacrifice the accumulation of PEP, which is a precursor of tyrosine [28]. *ptsG* and *crr* are the two components of PTS. However, after their deletion, the titer of (2*S*)-naringenin decreased to 137 mg/l, and the titer of *p*-coumaric acid and tyrosine increased to 558 mg/l and 590 mg/l, respectively (Fig. 5A). Such results indicate that too much tyrosine accumulation probably repressed the production of (2*S*)-naringenin for unknown reasons. In previous study, Gu *et al*. showed that the knockout of *pykF* could significantly enhance the metabolic flux of tyrosine biosynthesis [29]. However, in this study, knocking out *pheA* and *pykF* significantly influenced cell growth, thus reducing (2*S*)-naringenin production.

### Fed-Batch Fermentation in a 5-L Bioreactor

To further improve the titer of (2*S*)-naringenin, the best producer Nar-17LM1 was used for fed-batch fermentation in a 5-L bioreactor. The pH was maintained at 7.0 throughout the fermentation process. We found that the glucose was depleted after 60 h fermentation at 25°C. Feeding extra glucose improved the titer of (2*S*)-naringenin, achieving the highest (2*S*)-naringenin production of 588 mg/l (Fig. 5B). However, (2*S*)-naringenin started to degrade after 84 h fermentation, and the *p*-coumaric acid accumulation started rapidly. This phenomenon probably represents the (2*S*)-naringenin consumption pathway that exists naturally in *E. coli*.

## Discussion

(2*S*)-Naringenin biosynthesis in *E. coli* has suffered from low production. The reported highest titer was 100.64 mg/l with glucose as the substrate [14]. Furthermore, an expensive inducer (isopropyl-β-d-thiogalactoside) was used in the fermentation process, which elevated the biosynthesis cost. To overcome these challenges, using our previously constructed dynamic regulation strain (Mut-17) [19], we systemically optimized the components of the fermentation medium and fermentation conditions, obtaining 391 mg/l (2*S*)-naringenin from 3 mM L-tyrosine. Furthermore, we released the tyrosine feedback inhibition by deleting *tyrR* and overexpressing *aroG*^fbr^ and *tyrA*^fbr^. The optimum strain Nar-17LM1 produced 588 mg/l of (2*S*)-naringenin from glucose, the highest titer in *E. coli*. Compared with *S. cerevisiae* and *Yarrowia lipolytica*, the other two commonly used hosts, *E. coli* exhibited a shorter fermentation process. The reported highest de novo production titer of (2*S*)-naringenin in *S. cerevisiae* and *Y. lipolytica* was 220 mg/l [30] and 898 mg/l [18], respectively. Although the (2*S*)-naringenin titer of Nar-17LM1 was lower than *Y. lipolytica*, the fermentation process was significantly shorter (84 h vs. 300 h), making Nar-17LM1 a suitable producer for (2*S*)-naringenin.

The de novo biosynthesis pathway of (2*S*)-naringenin usually expresses more than 10 genes. Incongruous expression of these genes in one host often results in low productivity or impaired cell growth [31]. Hence, co-culture strategies were established in recent years to split the long pathways into different strains for reducing cell burden. To do so, Zhang *et al*. split the (2*S*)-naringenin de novo synthesis pathway into two parts: tyrosine synthesis pathway that uses D-xylose as the substrate, and (2*S*)-naringenin synthesis pathway that uses tyrosine as the substrate [32]. Tyrosine and (2*S*)-naringenin synthesis pathways were overexpressed in *E. coli* and *S. cerevisiae*, respectively. After a series of optimizations, they obtained 21.16 mg/l (2*S*)-naringenin. Likewise, Ganesan *et al*. used a *p*-coumaric acid-producing strain and a (2*S*)-naringenin-producing strain in a co-culture strategy [33] and obtained 40 mg/l (2*S*)-naringenin. However, it is difficult to fine-tune a co-culture system in the fermentation process, leading to low productivity. The transportation of intermediates, such as tyrosine and *p*-coumaric acid, further reduces the synthesis efficiency since such intermediates need to transfer to other cells for (2*S*)-naringenin biosynthesis. Here, we used a dynamic regulated strain as a platform for de novo production of (2*S*)-naringenin. This strain is capable of automatically regulating its metabolic flux to balance cell growth and (2*S*)-naringenin production, and thus, reduce the burden caused by the expression of a long metabolic pathway. As the point of comparison with other dynamic regulated strains, the (2*S*)-naringenin titer of Nar-17LM1 was 3.7- and 4.3-fold of the reports of Lv *et al*. [34] and Dinh *et al*. [35, 36], respectively.

In the fermentation process, we found that organic nitrogen sources, such as yeast extract and peptone, promoted cell growth but reduced (2*S*)-naringenin production (Fig. 2). One possible reason was that too fast cell growth would exhaust the cell resources that should be used to express the biosynthesis pathway of (2*S*)-naringenin. Hence, a more flexible strategy needs to be developed to distribute cell resources between cell growth and (2*S*)-naringenin production. On the other hand, the fermentation temperature significantly influenced the production of (2*S*)-naringenin and *p*-coumaric acid. A higher temperature (30°C) was suitable for *p*-coumaric acid production, but a lower temperature (20°C) was suitable for (2*S*)-naringenin production (Fig. 3A). This phenomenon was probably caused by differences in the optimum catalyzation temperature between TAL and the downstream enzymes (4CL, CHS, and CHI). In this regard, future research is needed for screening or engineering TAL to fit the catalyzation temperature of the downstream enzymes. Taken together, the results presented in this study indicate that *E. coli* is still a superior host for producing (2*S*)-naringenin or other flavonoids.

## Figures and Tables

**Table 1:** Strains and plasmids used in this study.

Strains and plasmids	Properties	Sources
Strains		
*E. coli* JM109	Wild type	This study
*E. coli* BL21 (DE3)	Wild type	This study
Mut-17	(2*S*)-Naringenin dynamic regulated strain which carrying pETM-PUTR_trxA_-TAL-PUTR_talB_-4CL, pCDM-P_ssrA_-UTR_rpsT_-CHS-PUTR_glpD_-CHI, pACM-P_fdeR_-FdeR-P_fdeA-17_-acpH-asacpT-asacpS, and pRSM-PadR-acs-ACC	[19]
Nar-17LL	pCDM-P_ssrA_-UTR_rpsT_-CHS-PUTR_glpD_-CHI was replaced by pCDM-P_ssrA_-UTR_rpsT_-*chs*-PUTR_glpD_-*chi*-P_*yjiY*_-*tyrA*^fbr^-P_*yjiY*_-*aroG*^fbr^ in Mut-17	This study
Nar-17HL	pCDM-P_ssrA_-UTR_rpsT_-CHS-PUTR_glpD_-CHI was replaced by pCDM-P_ssrA_-UTR_rpsT_-*chs*-PUTR_glpD_-*chi*-P_*glpD*_-*tyrA*^fbr^-P_*yjiY*_-*aroG*^fbr^ in Mut-17	This study
Nar-17HM	pCDM-P_ssrA_-UTR_rpsT_-CHS-PUTR_glpD_-CHI was replaced by pCDM-P_ssrA_-UTR_rpsT_-*chs*-PUTR_glpD_-*chi*-P_*glpD*_-*tyrA*^fbr^-P_*talB*_-*aroG*^fbr^ in Mut-17	This study
Nar-17LM	pCDM-P_ssrA_-UTR_rpsT_-CHS-PUTR_glpD_-CHI was replaced by pCDM-P_ssrA_-UTR_rpsT_-*chs*-PUTR_glpD_-*chi*-P_*yjiY*_-*tyrA*^fbr^-P_*talB*_-*aroG*^fbr^ in Mut-17	This study
Nar-17LM1	*tyrR* was knockout in Nar-17LM	This study
Nar-17LM2	*tyrR*, *pstG*, and *crr* were knockout in Nar-17LM	This study
Nar-17LM3	*tyrR*, *pstG*, *crr*, and *pheA* were knockout in Nar-17LM	This study
Nar-17LM4	*tyrR*, *pstG*, *crr*, *pheA*, and *pykF* were knockout in Nar-17LM	This study
Plasmids		
pETM-PUTR_trxA_-TAL-PUTR_talB_-4CL	Amp^r^, pETM6 backbone, the expression of *tal* and *4cl* was controlled by PUTR_trxA_ and PUTR_talB_, respectively.	[7]
pCDM-P_ssrA_-UTR_rpsT_-CHS-PUTR_glpD_-CHI	Str^r^, pCDM4 backbone, the expression of *chi* and *chs* was controlled by P_ssrA_-UTR_rpsT_ and PUTR_glpD_, respectively.	[7]
pACM-P_fdeR_-FdeR-P_fdeA-17_-acpH-asacpT-asacpS	Cm^r^, pACM4 backbone, P_fdeR_ and P_fdeA-17_ controlled the expression of *fdeR* and *acpH*, respectively. P_fdeA_ controlled the expression of as*acpT* and as*acpS*.	[19]
pRSM-PadR-acs-*ACC*	Kan^r^, pRSM3 backbone, P_cspA_ and P_padC_ controlled the expression of *padR* and *acs, ACC*, respectively.	[19]
pMD-*tyrA*^fbr^-*aroG*^fbr^	Amp^r^, T-vector pMDTM19 (Simple) carrying *tyrA*^fbr^ and *aroG*^fbr^	[14]
pMD-P_*yjiY*_-*tyrA*^fbr^-*aroG*^fbr^	Amp^r^, pMD-*tyrA*^fbr^-*aroG*^fbr^ backbone, P_*yjiY*_ controlled the expression of *tyrA*^fbr^	This study
pMD-P_*glpD*_-*tyrA*^fbr^-*aroG*^fbr^	Amp^r^, pMD-*tyrA*^fbr^-*aroG*^fbr^ backbone, P_*glpD*_ controlled the expression of *tyrA*^fbr^	This study
pMD-P_*yjiY*_-*tyrA*^fbr^-P_*talB*_-*aroG*^fbr^	Amp^r^, pMD-P_*yjiY*_-*tyrA*^fbr^-*aroG*^fbr^ backbone, P_*talB*_ controlled the expression of *aroG*^fbr^	This study
pMD-P_*yjiY*_-*tyrA*^fbr^-P_*yjiY*_-*aroG*^fbr^	Amp^r^, pMD-P_*yjiY*_-*tyrA*^fbr^-*aroG*^fbr^ backbone, P_*yjiY*_ controlled the expression of *aroG*^fbr^	This study
pMD-P_*glpD*_-*tyrA*^fbr^-P_*yjiY*_-*aroG*^fbr^	Amp^r^, pMD-P_*glpD*_-*tyrA*^fbr^-*aroG*^fbr^ backbone, P_*yjiY*_ controlled the expression of *aroG*^fbr^	This study
pMD-P_*glpD*_-*tyrA*^fbr^-P_*talB*_-*aroG*^fbr^	Amp^r^, pMD-P_*glpD*_-*tyrA*^fbr^-*aroG*^fbr^ backbone, P_*talB*_ controlled the expression of *aroG*^fbr^	This study
pCDM-P_ssrA_-UTR_rpsT_-*chs*-PUTR_glpD_-*chi*-P_*yjiY*_-*tyrA*^fbr^-P_*talB*_-*aroG*^fbr^	Str^r^, pCDM-P_ssrA_-UTR_rpsT_-CHS-PUTR_glpD_-CHI backbone, P_*yjiY*_ and P_*talB*_ controled the expression of *tyrA*^fbr^ and *aroG*^fbr^, respectively.	This study
pCDM-P_ssrA_-UTR_rpsT_-*chs*-PUTR_glpD_-*chi*-P_*yjiY*_-*tyrA*^fbr^-P_*yjiY*_-*aroG*^fbr^	Str^r^, pCDM-P_ssrA_-UTR_rpsT_-CHS-PUTR_glpD_-CHI backbone, P_*yjiY*_ and P_*yjiY*_ controled the expression of *tyrA*^fbr^ and *aroG*^fbr^, respectively.	This study
pCDM-P_ssrA_-UTR_rpsT_-*chs*-PUTR_glpD_-*chi*-P_*glpD*_-*tyrA*^fbr^-P_*yjiY*_-*aroG*^fbr^	Str^r^, pCDM-P_ssrA_-UTR_rpsT_-CHS-PUTR_glpD_-CHI backbone, P_*glpD*_ and P_*yjiY*_ controled the expression of *tyrA*^fbr^ and *aroG*^fbr^, respectively.	This study
pCDM-P_ssrA_-UTR_rpsT_-*chs*-PUTR_glpD_-*chi*-P_*glpD*_-*tyrA*^fbr^-P_*talB*_-*aroG*^fbr^	Str^r^, pCDM-P_ssrA_-UTR_rpsT_-CHS-PUTR_glpD_-CHI backbone, P_*glpD*_ and P_*talB*_ controled the expression of *tyrA*^fbr^ and *aroG*^fbr^, respectively.	This study

**Table 2:** Primers used in this study.

Primers	Sequence (from 5′ to 3′)*
P_*yjiy*_-*aroG*-F	GAAATAATTTTGTTTAACTTTAATAAGGAGATATAGCGGCCGCAAATAACCACTCAGTTATTTACCTTAC
P_*yjiy*_-*aroG*-R	CGTAAATCGTCGTTCTGATAATTCATCCCGGGAGTAAAACCTGGCATGTATTGAT
P_*yjiy*_-*tyrA*-F	TAAAAGCGCGTCGCGGGTAACTGCAGAAATAACCACTCAGTTATTTACCTTAC
P_*yjiy*_-*tyrA*-R	CGTAATGCGGTCAATTCAGCAACCATGGAGTAAAACCTGGCATGTATTGAT
P_*talB*_-*aroG*-F	GAAATAATTTTGTTTAACTTTAATAAGGAGATATAGCGGCCGCCCTGGCGATAACCGTCTT
P_*talB*_-*aroG*-R	CGTAAATCGTCGTTCTGATAATTCATCCCGGGGATAGTATTTCTCTTTAAACAGCTTGT
P_*glpD*_-*tyrA*-F	TAAAAGCGCGTCGCGGGTAACTGCAGTCACTCTAAAATGTTTTTTCAATGT
P_*glpD*_-*tyrA*-R	CGTAATGCGGTCAATTCAGCAACCATGGGCTGCCCTCATTCACTTTC
*aroG*-F	CAATTCCCCTGTAGAAATAATTTTGTTTAAC
*aroG*-R	GTAACTCTTTGATTTCTTTGATGCGTAAATC
*tyrA*-F	CTGTTACGTCAACTGGCGAATGC
*tyrA*-R	TATCGACTTCATCAATTTGATCGCG
*tyrA*-*aroG*-F	TGATCTTTTCTACTGAACCGCTCTAGACCCCTGTAGAAATAATTTTGTTTAACTTTAATA
*tyrA*-*aroG*-R	CGGATGTGATAGCCAATGGATCCGACCATGATTACGCCAAGTTTGC
